# A Novel Case of Trigger Finger Caused by A1 Pulley Calcification Successfully Treated With Ultrasound-Guided Prolotherapy: A Case Report and Literature Review

**DOI:** 10.7759/cureus.98189

**Published:** 2025-11-30

**Authors:** Yonghyun Yoon, Ji Hyo Hwang, Jaehyun Shim, Rowook Park, Jaeyoung Lee, King Hei Stanley Lam

**Affiliations:** 1 Orthopedic Surgery, Hallym University Kangnam Sacred Heart Hospital, Seoul, KOR; 2 Orthopedics, Incheon Terminal Orthopedic Surgery Clinic, Incheon, KOR; 3 Neurosurgery, Chungdammadi Neurosurgery Clinic, Seoul, KOR; 4 Rehabilitation Medicine, Sae Yonsei Rehabilitation Clinic, Seoul, KOR; 5 Faculty of Medicine, The Chinese University of Hong Kong, New Territories, HKG; 6 Faculty of Medicine, The University of Hong Kong, Hong kong, HKG; 7 The Board of Clinical Research, The Hong Kong Institute of Musculoskeletal Medicine, Kowloon, HKG

**Keywords:** a1 pulley, calcific tendinitis, calcific tendinopathy, prolotherapy, stenosing tenosynovitis, trigger finger, ultrasound-guided intervention

## Abstract

Trigger finger is commonly attributed to thickening of the A1 pulley or flexor tendons. Although calcific tendinitis is well recognized in regions such as the shoulder, calcification within the A1 pulley itself is extremely rare and seldom reported as a primary cause of triggering. We describe a 43-year-old woman who presented with painful triggering of the right little finger, accompanied by morning stiffness, snapping, and focal tenderness with warmth over the volar metacarpophalangeal (MCP) joint. Radiographs and high-resolution ultrasonography revealed a calcific deposit in the superficial layer of the A1 pulley, with surrounding hypervascularity on Doppler imaging. The patient underwent three sessions of ultrasound-guided prolotherapy in conjunction with physiotherapy and night splinting, which led to complete resolution of pain, stiffness, and triggering. This case identifies A1 pulley calcification as a rare but distinct etiology of trigger finger and highlights the diagnostic utility of ultrasonography in detecting this lesion. Moreover, it demonstrates that ultrasound-guided prolotherapy can provide an effective, minimally invasive alternative to surgical release, particularly when standard conservative management fails. Ultrasound-guided prolotherapy was selected as the primary intervention, instead of corticosteroid injection (CI), given its potential to promote tissue repair and resorption of calcific deposits, which are less effectively addressed by corticosteroids whose effect is primarily anti-inflammatory. Symptom improvement was observed after each biweekly session, with complete resolution achieved four weeks after the initial intervention. In addition to pain reduction documented by the Visual Analog Scale (VAS), the patient experienced the elimination of morning stiffness and snapping and restoration of full finger range of motion (ROM) following the final prolotherapy session.

## Introduction

Trigger finger, or stenosing tenosynovitis, is a prevalent condition characterized by a painful snapping or locking of a digit during flexion and extension. It most commonly affects women in their 40s and 50s [1], with a predilection for the ring finger and thumb [2]. The pathophysiology typically involves a disproportion between the flexor tendon and its pulley system, primarily due to thickening of the A1 pulley or the tendon itself [3]. It is often associated with systemic conditions such as hypothyroidism, diabetes mellitus, hyperlipidemia, and rheumatoid arthritis [3].

Calcific tendinitis, characterized by hydroxyapatite crystal deposition within soft tissues, typically progresses through formative, resting, and resorptive stages. During the resorptive phase, macrophage- and vascular-driven inflammations contribute to pain and swelling. While this process is well documented in regions such as the rotator cuff, its occurrence within the compact fibro-osseous structure of the A1 pulley is exceptionally uncommon. In rare cases, calcific hydroxyapatite deposition may occur within the A1 pulley itself, resulting in triggering through a distinct mechanical obstruction mechanism. This pathology is seldom reported in the literature [4,5], especially as a primary cause of digit locking. The deposition of calcium crystals creates a focal, rigid barrier to tendon gliding, inciting dynamic friction and sometimes localized inflammation. Although calcific tendinitis is well recognized in larger joints, its occurrence in the A1 pulley represents a unique clinical challenge.

The diagnostic process has been revolutionized by high-resolution ultrasonography (US), which allows for dynamic, real-time visualization of the pulley system and tendons. Key sonographic findings include A1 pulley thickening, flexor tendon thickening, nodule formation, and volar plate abnormalities [6,7]. Importantly, the US provides the resolution necessary to differentiate calcific deposits from adjacent sesamoid bone and tendon structures, making it uniquely suited for detecting rare intrapulley calcifications.

First-line treatments include conservative measures such as activity modification, splinting, and physiotherapy [8]. When these fail, corticosteroid injections (CSIs) are the mainstay of interventional treatment [9]. For refractory cases, surgical options like open or US-guided A1 pulley release are highly effective [7]. However, alternative minimally invasive treatments like prolotherapy [10] and hydrodissection [11] are gaining interest. In cases involving calcific pathology, corticosteroids may provide only temporary symptomatic relief because their effect is primarily anti-inflammatory and does not facilitate the removal of calcific material. In contrast, prolotherapy has the potential to induce a controlled inflammatory response that promotes macrophage recruitment, fibroblast activation, and, potentially, enhanced resorption of hydroxyapatite deposits, mechanisms that may be particularly advantageous for calcific lesions.

Given the mechanical nature of calcific A1 pulley pathology, prolotherapy may exert a therapeutic effect through the induction of a targeted local healing response, in contrast to the primarily anti-inflammatory action of CIs. This approach is uniquely suited to promote resorption of calcific material and restore pulley integrity. Despite scattered anecdotal reports and success in other anatomical regions [12], there is a paucity of data regarding prolotherapy’s utility for calcific pathology in the hand.​

We report a unique case of trigger finger caused by a calcific deposit within the A1 pulley, successfully managed with US-guided prolotherapy. To our knowledge, this is the first report to describe both the sonographic identification of this specific pathology and its successful resolution with prolotherapy.

## Case presentation

A 43-year-old, right-handed woman presented with a four-week history of pain, morning stiffness, and a painful snapping sensation in her right little finger. She had no history of diabetes, thyroid dysfunction, or inflammatory arthritis.

On physical examination, there was marked tenderness and localized warmth over the volar aspect of the fifth metacarpophalangeal (MCP) joint. Active flexion and extension reproduced a painful snapping phenomenon.

**Figure 1:**
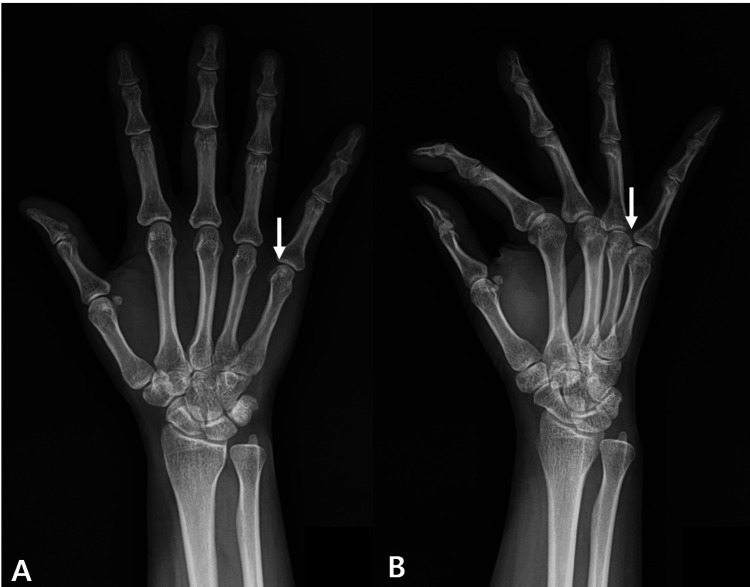
Calcifications adjacent to the sesamoid bone can be seen on X-ray. Calcifications distinct from the sesamoid are observed in the (A) AP view (white arrow) and (B) oblique view (white arrow).

Plain radiographs demonstrated a faint calcific density adjacent to the MCP joint, distinct from the sesamoid bone (Figure 1).

**Figure 2:**
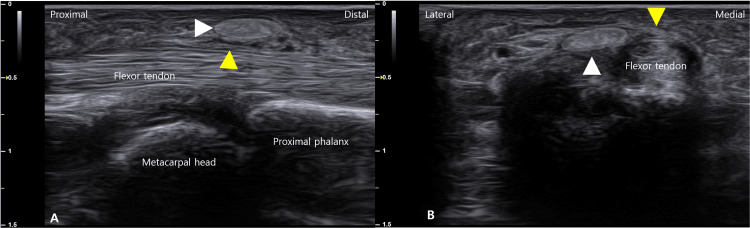
Long- and short-axis ultrasonography of the A1 pulley. In the long-axis view, a hyperechoic calcific deposit (white arrowhead, white-shaded area) is visualized above the A1 pulley (yellow arrowhead) (A), and in the short-axis view, the calcific focus (white arrowhead, white shaded area) is observed on the lateral side of the A1 pulley (yellow arrowhead) (B).

**Figure 3:**
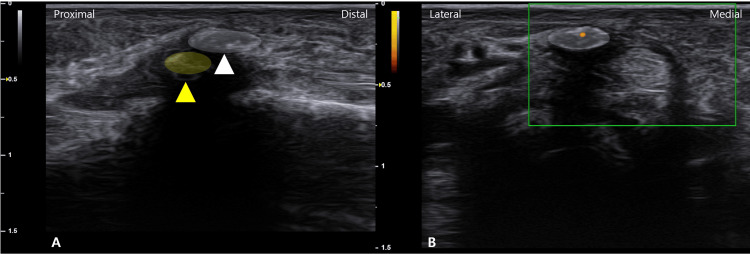
Ultrasound findings of the calcification of the A1 pulley. (A) Calcification (white arrowhead, white shaded area) is observed separately from the sesamoid bone (yellow arrowhead) on a long-axis image. (B) Signal changes are observed on power Doppler.

High-resolution US revealed a hyperechoic calcific deposit within the superficial layer of the A1 pulley (Figures 2A, 2B), clearly distinguishable from the underlying flexor tendon and adjacent sesamoid bone (Figure 3A). Power Doppler imaging showed mild hyperemia in the surrounding soft tissue, supporting the presence of active inflammation (Figure 3B). No significant tendon nodule or additional pulley thickening was observed.

Intervention and outcome

A treatment plan for US-guided prolotherapy was proposed and accepted by the patient. Under real-time guidance using a high-frequency linear transducer, a 25-gauge needle was carefully advanced to the periphery of the hyperechoic calcific deposit within the A1 pulley. The prolotherapy solution, consisting of 10% dextrose and 0.2% lidocaine, was injected circumferentially around the deposit to induce a focal healing response and facilitate potential resorption of calcific material.

The patient received three such prolotherapy injections at two-week intervals. Adjunctive modalities included a structured physiotherapy protocol, comprising passive stretching and targeted ROM exercises for the affected digit, delivered three times weekly for two weeks, and nocturnal splinting in a neutral position.

**Figure 4:**
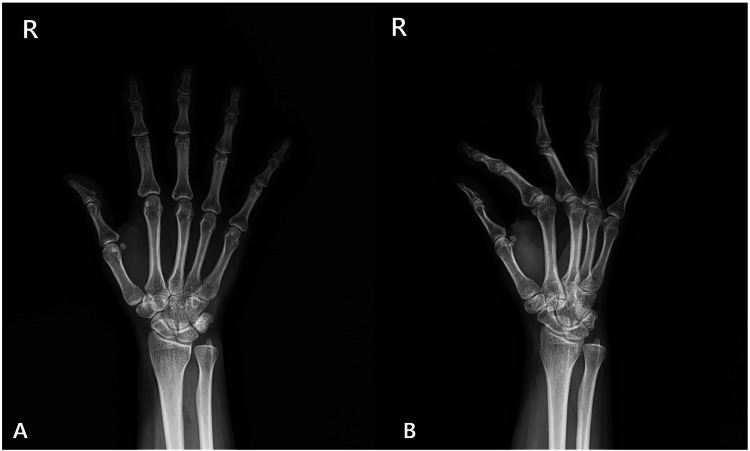
After six weeks, follow-up hand radiographs in the anteroposterior (A) and oblique (B) views demonstrated interval resorption of the calcific deposit.

Following the final prolotherapy session, the patient experienced complete resolution of morning stiffness, triggering, and local pain. Physical examination documented restoration of full active ROM and absence of palpable snapping or tenderness. Pain intensity, as measured by the Visual Analog Scale (VAS), improved from 7/10 at baseline to 0/10 at the four-week follow-up. Follow-up radiographs obtained at six weeks confirmed near-complete resolution of the calcific deposit, consistent with the patient’s symptomatic improvement (Figure 4).

This case report is based on a retrospective review of medical records. All treatments and evaluations were performed as part of routine clinical care. The study utilized fully anonymized data, with all personal identifiers removed from the dataset. Therefore, this research met the criteria for exemption from review by an Institutional Review Board (IRB).

## Discussion

This case is noteworthy for two primary reasons. First, it identifies A1 pulley calcification as a direct and rare etiology for trigger finger, a pathology seldom described in the literature. A search of existing publications yields only a handful of reports. The most comparable case, reported by Seiler and Kerwin, described a calcification within the A1 pulley that was successfully treated with surgical excision [5]. A more recent case from Kelly and Maxwell reported a calcific deposit near the A1 pulley causing trigger finger; however, a critical distinction is that their case involved calcification within the flexor digitorum profundus tendon immediately adjacent to the pulley, rather than a deposit intrinsic to the pulley ligament itself [4]. This highlights that our presented case is, to the best of our knowledge, the first to utilize high-resolution US to clearly identify and target a calcific deposit located specifically within the substance of the A1 pulley. The US clearly delineated the hyperechoic focus above the pulley structure, distinct from both the flexor tendon and the adjacent sesamoid bone, thereby confirming it as the primary obstructing pathology.

Second, and more significantly, this report presents US-guided prolotherapy as a novel and effective non-surgical treatment for this specific condition. While prolotherapy, the injection of an irritant solution to stimulate the body's natural wound-healing mechanisms, is increasingly used for common tendinopathies [13], its application for A1 pulley calcific tendinitis has not been previously documented. The success in this case suggests a plausible dual mechanism of action. The injected dextrose solution is hypothesized to incite a controlled, localized inflammatory cascade [14,15]. This response may recruit macrophages and other inflammatory cells that can phagocytose and resorb the calcium hydroxyapatite crystals. Simultaneously, this process promotes fibroblast proliferation and collagen deposition, leading to the reinforcement and strengthening of the compromised pulley ligament. This mechanistic approach provides a compelling non-surgical alternative that addresses the root cause of the problem.

Recent systematic reviews have supported the role of dextrose prolotherapy in stimulating localized repair and facilitating the resorption of calcific deposits in tendons and ligaments [16]. In contrast, CSIs, the current interventional mainstay for typical trigger finger, exert a potent anti-inflammatory effect. However, CSIs may provide only transient relief when confronted with a predominantly mechanical pathology such as calcific impingement, and they carry the risk of connective tissue weakening. Therefore, prolotherapy may be more effective in treating calcific lesions due to its ability to trigger active repair.

Adjunctive physiotherapy, including passive stretching and ROM exercises, was incorporated to support recovery by reducing stiffness and optimizing tendon-pulley gliding. Although physiotherapy likely contributed to maintaining motion and minimizing discomfort, the complete and rapid resolution of symptoms arguably resulted from direct US-guided prolotherapy of the calcific deposit.

The main limitations of this report are inherent in its design as a single case report. The lack of long-term follow-up imaging prevents definitive confirmation of the calcification's complete dissolution, and the outcomes are based on a single patient. Future research should prioritize controlled studies with serial imaging to validate prolotherapy's efficacy and clarify long-term outcomes for calcific pulley pathologies.

## Conclusions

This case expands the differential diagnosis for trigger finger to include A1 pulley calcific tendinitis. It underscores the critical role of the US in identifying atypical underlying pathologies. Moreover, it demonstrates that US-guided prolotherapy can be a viable, minimally invasive, and effective treatment modality for this rare condition, offering a potential alternative to surgery. Physicians should be aware of this entity, particularly in cases that are refractory to standard CIs.
